# Current Landscape of Compression Products for Treatment of Postural Orthostatic Tachycardia Syndrome and Neurogenic Orthostatic Hypotension

**DOI:** 10.3390/jcm13237304

**Published:** 2024-12-01

**Authors:** Kishen Mitra, Sameer Kunte, Sara Taube, William Tian, Eric Richardson, Camille Frazier-Mills, Marat Fudim

**Affiliations:** 1 Department of Biomedical Engineering, Duke University, Durham, NC 27708, USA; 2 Duke University School of Medicine, Durham, NC 27710, USA; 3 Division of Cardiology, Duke University Medical Center, Durham, NC 27710, USAmarat.fudim@duke.edu (M.F.)

**Keywords:** postural orthostatic tachycardia syndrome (POTS), neurogenic orthostatic hypotension (nOH), compression garments, product features, survey study

## Abstract

**Background/Objectives**: Patients with postural orthostatic tachycardia syndrome (POTS) or neurogenic orthostatic hypotension (nOH) experience vertigo, confusion, and syncope. Compression garments help reduce venous pooling in these patients, thereby increasing cardiac output. We aimed to determine end-user opinions of compression products intended to alleviate symptoms for POTS and nOH. **Methods**: This was a survey study sampling participants diagnosed with POTS or nOH. The data collected included demographics, medical history, and compression garments previously used. The participants rated their most frequently used garment across comfort, aesthetic appeal, ease of use, durability, cost-effectiveness, efficacy, and consistency on the Likert scale (1–5). One-way ANOVA was used to compare the design criteria ratings across garments. For all tests α = 0.05. **Results**: Of the 330 POTS and 28 nOH participants surveyed (mean age 37.9, mean BMI 27.5, 95.0% women, 90.5% White), 354 (98.9%) reported trying at least one compression garment since their diagnosis. The majority of participants reported using leg compression most frequently rather than shapewear or abdominal compression (65.4% vs. 20.1% vs. 13.4%, respectively). Approximately 67.0% of participants tried multiple product types. Shapewear was reported to have greater concealability compared to abdominal or leg compression garments (mean 3.43 vs. 2.90 vs. 2.91, respectively; *p* < 0.01). Shapewear and abdominal compression were rated to be less comfortable compared to leg compression (2.67 vs. 2.94 vs. 3.05, respectively; *p* = 0.03). **Conclusions**: The existing compression products do not fully meet needs of individuals with POTS or nOH, as evidenced by participant ratings on multiple domains. There is potential consumer demand for novel adjustable abdominal compression garments that are low-profile and comfortable when disengaged.

## 1. Introduction

Postural orthostatic tachycardia syndrome (POTS) is a chronic condition that has orthostatic intolerance as its primary symptom. Patients may experience symptoms like fatigue, nausea, cognitive dysfunction, and loss of consciousness [1]. An estimated 3 million individuals in the United States have POTS [2,3]. POTS patients tend to be between the ages of 15 and 50 and are five times more likely to be women [4,5]. The pathophysiology of POTS relates to impaired autonomic responses [6]. When healthy individuals change body position, the heart and vasculature compensate for shifts in blood volume due to gravity by increasing heart rate, cardiac contractility, and vascular tone [7]. The result is a maintenance of perfusion to the brain and other vital organs. Patients with POTS typically have underlying hypovolemia, deconditioning, neuroendocrine dysfunction, and neuropathy [6,8,9,10]. These factors lead to impaired adaptation to changes in body position and the reflex increase in heart rate that defines POTS [6].

Neurogenic orthostatic hypotension (nOH) is another form of dysautonomia characterized by a failure to appropriately regulate blood pressure upon standing, leading to a significant drop in blood pressure due to inadequate norepinephrine release and impaired vasoconstriction of peripheral vessels [11]. nOH is an orphan condition as it effects fewer than 200,000 individuals in the United States [12]. This condition is often associated with neurodegenerative diseases such as Parkinson’s disease and multiple system atrophy. Patients with nOH experience similar symptom profiles to patients with POTS.

The treatment of POTS and nOH includes a multifaceted approach of behavioral modification, non-pharmacological therapy, and pharmacological therapy [1,7]. Behavioral modification includes increasing fluid intake and sodium consumption, regular exercise, avoidance of substances that worsen symptoms like alcohol, and caution when changing body position. Non-pharmacological therapies include wearing compression garments on the legs and abdomen. The initial management should focus on behavioral modifications and non-pharmacological therapies before considering pharmacotherapy [13]. Pharmacotherapy may be reserved for moderate to severe cases that do not respond to behavioral modification and non-pharmacological therapies due to the side effects associated with commonly prescribed drugs, as well as variability in patient responses [10,14,15,16].

Compression garments are known to reduce symptoms in POTS and nOH [17,18,19,20]. Patients with POTS and nOH typically have excessive venous pooling in the legs and abdomen due to inadequate vascular tone [21]. Compressive garments provide extracorporeal pressure to promote blood flow back to the heart to improve cardiac output, thereby reducing reflex tachycardia and orthostatic symptoms [22].

Despite the documented efficacy of compression therapy, there is a paucity of information regarding end-user opinions of these products. Psychological and design-related factors associated with wearing compression garments can affect adherence and, in turn, treatment outcomes [23]. This gap in understanding patient perspectives motivated our group to investigate user experiences and satisfaction with existing compression products in the context of POTS and nOH management.

## 2. Materials and Methods

### 2.1. Study Participants

Patients with POTS and nOH were recruited from social media, online patient support groups, and the Duke Syncope and Dysautonomia Clinic. Inclusion criteria were a self-reported physician diagnosis of POTS or nOH and age 18 or older. Exclusion criteria were diagnosis of autonomic disorders other than POTS or nOH. The participants were required to have a diagnosis of POTS or nOH made either via a tilt table test or a 10 min stand test performed at a doctor’s office.

All study participants consented to participate in the study. The study was approved by the Institutional Review Board at Duke University Health System (approved 8 March 2023).

### 2.2. Study Design

#### 2.2.1. Forms of Compression

**Figure 1:**
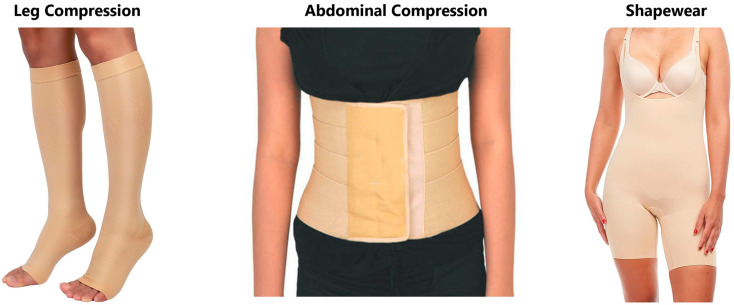
Visual representation of the three compression garment categories evaluated in this study.

The existing products were segmented into the categories of medical leg compression, medical abdominal compression, and shapewear (Figure 1). Each of the forms of compression garments serve a distinct functionality.

Medical leg compression refers to garments specifically designed to exert graduated pressure on the leg. These garments can be engineered to apply higher pressure at the ankle, gradually decreasing as they move up the leg. Example garments include knee, thigh, and waist high stockings, as well as compression socks. The pressure exerted by stockings usually ranges from 20 to 40 mmHg, which is clinically validated for therapeutic effectiveness [24]. As per international classification of pressure exertion intensity for treatment of vascular medical conditions, it is Class I (light) up to 20 mmHg; Class II (moderate) 20–30 mmHg; Class III (firm) 30–40 mmHg; and Class IV (extra firm) 40 mmHg and above [25,26].

Unlike leg compression, medical abdominal compression garments may not have a graduated pressure design but instead provide consistent support to the entire abdomen area. Medical abdominal compression provides a similar magnitude of pressure (20 to 40 mmHg) to medical leg compression. Furthermore, the same classification of pressure exertion can be applied to abdominal compression [22]. Example garments include compression girdles, abdominal binders, and compression vests

Shapewear is primarily designed for aesthetic purposes to smooth out the body’s contours. The pressure exerted by shapewear is typically lower (10 to 20 mmHg), less consistent, and non-graduated. This pressure is not standardized and does not follow the guidelines for medical compression garments. Shapewear can include items like bodysuits and camisoles that are intended to be worn under clothing.

#### 2.2.2. Survey

All participants completed a survey consisting of four sections. The first section collected demographic information and medical history. In the second section, patients selected which form(s) of compression garments they have previously used and which garment they have used the most frequently. Data on the daily wear duration of compression garments were not collected.

In the third section, patients rated the compression garment they used the most frequently across a series of product features on the Likert scale of 1 to 5 (Table 1).

In the final section, participants were asked to select whether each of the following are perceived to help reduce their symptoms: fluid and salt intake, exercise, compression garments, or pharmacotherapy. Participants were not required to detail the specific medications that were most effective.

### 2.3. Statistical Analysis

Pearson’s Chi-squared Tests were performed to compare gender, ethnicity, type of compression garment used, most used compression garment, and efficacy of treatment modalities between participants diagnosed with POTS and nOH. Welch T-Tests were performed to compare age and BMI between participants diagnosed with POTS and nOH.

One-way ANOVA was used to compare the product features ratings across garments. A significance level was set at *p* < 0.05. Post hoc testing was performed when applicable to discover which specific type of compression products were significantly different from one another. All statistical analyses were performed using RStudio (RStudio, PBC, Version 4.2.1).

## 3. Results

The participating 330 POTS patients had a mean age of 36.9 ± 11.4 (range 18–76 years) and the participating 28 nOH patients had a mean age of 49.6 ± 14.8 (range 21–78 years) (Table 2). Across the entire cohort, the mean BMI was 27.5 ± 7.60 (range 16.4–52.1). Furthermore, 95.0% of participants were women, and 90.5% of participants were White.

### 3.1. Forms of Compression Garments

**Figure 2:**
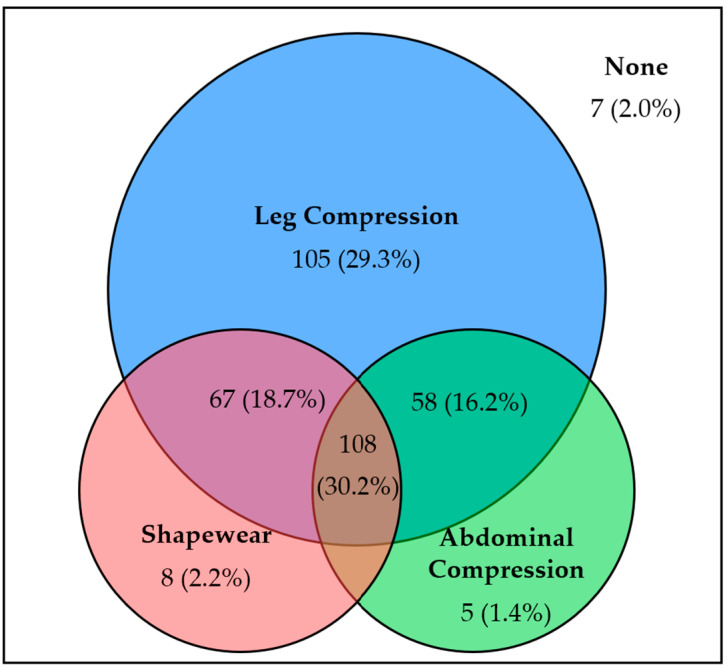
Venn diagram illustrating the number of participants that have used either one form of compression garment or multiple forms of compression garments.

Three hundred and fifty-one (98.0%) participants reported trying at least one compression garment since their diagnosis. Among the 118 (33.0%) participants who only used one form of compression garment, leg compression was the most common (Figure 2). Sixty-seven (18.7%) participants used shapewear and leg compression. Fifty-eight (16.2%) participants used abdominal compression and leg compression. One hundred and eight (30.2%) participants used all three forms of compression garments.

In total, 183 (51.1%) of participants used shapewear and 171 (47.8%) of participants used abdominal compression garments (Table 2). Shapewear was slightly more popular amongst POTS patients compared to nOH patients (51.5% vs. 46.4%; *p* = 0.60). Abdominal compression garments were more popular amongst nOH patients compared to POTS patients (60.7% vs. 46.7%; *p* = 0.15). Leg compression was used by 338 (94.4%) participants at some point since diagnosis.

Of the participants who tried at least one form of compression, 234 (65.4%) participants reported using leg compression most frequently. Seventy-two (20.1%) participants reported using shapewear most frequently. Forty-eight (13.4%) participants reported using abdominal compression most frequently.

### 3.2. Product Feature Ratings

**Figure 3:**
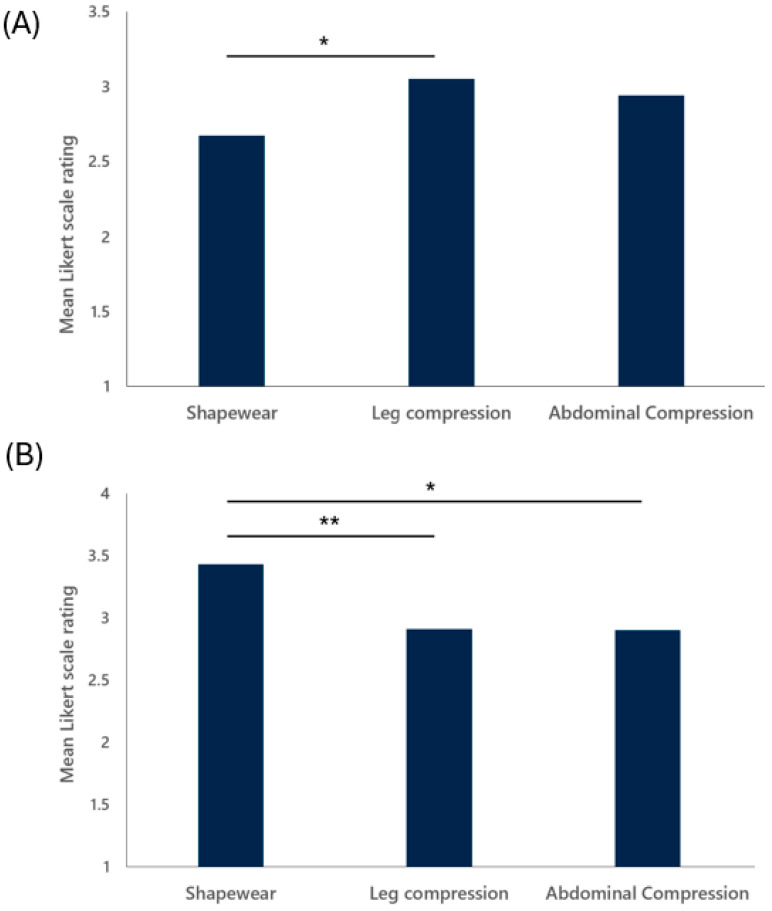
Participant ratings comparison for (**A**) comfort and (**B**) concealability product features. * *p* < 0.05, ** *p* < 0.01.

POTS and nOH patients rated shapewear to be significantly less comfortable compared to leg compression (mean 2.67 vs. 3.05, respectively; *p* = 0.02) (Figure 3A). Abdominal compression was rated to be less comfortable than leg compression without reaching statistical significance (mean 2.94 vs. 3.05, respectively; *p* = 0.78). Conversely, shapewear were reported to have greater concealability compared to abdominal or leg compression garments (mean 3.43 vs. 2.90 vs. 2.91, respectively; *p* < 0.01) (Figure 3B).

Participants rated abdominal compression garments to be significantly more cost-effective than shapewear and leg compression garments (mean 3.15 vs. 2.90 vs. 2.73, respectively; *p* = 0.028) (Appendix A). Cost-effectiveness ratings in this study were subjective measures influenced by individual value judgments of a product’s worth, considering both its cost and perceived health benefits. All three forms of compression garments were rated to have comparable levels of aesthetic appeal, ease of use, durability, and consistency (Table A1).

**Figure 4:**
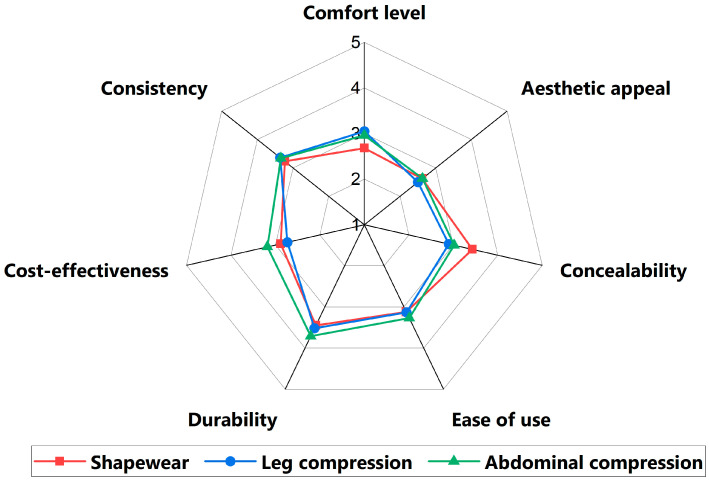
Product feature rating aggregated results.

The mean Likert scale ratings across all features scored below 4 (Figure 4). Unmet needs were especially apparent in the areas of aesthetic appeal, comfort, and ease of use.

### 3.3. Different Treatment Approaches

Fluid and salt intake was perceived to effectively mitigate symptoms in 209 (58.4%) participants (Table 3). Pharmacotherapy was perceived to effectively mitigate symptoms in 153 (42.7%) participants. Exercise, such as aerobic conditioning, was perceived to effectively mitigate symptoms in 76 (21.2%) participants. Compression garments were perceived to effectively mitigate symptoms in 32 (8.9%) participants.

These findings suggest that the existing compression garments may provide some temporary relief, but they do not significantly improve the overall quality of life for most POTS and nOH patients.

## 4. Discussion

### 4.1. Main Findings

In this study, we found that leg compression garments were used more than abdominal compression garments amongst POTS and nOH patients. Leg compression garments are often more accessible and widely accepted among patients. They are commonly available in various styles and pressures, potentially making them easier to incorporate into daily life. Familiarity associated with leg compression garments may contribute to their preference. Many patients find leg compression stockings to be less intrusive than abdominal compression devices, which can feel restrictive and uncomfortable [27].

However, there is some evidence that abdominal compression is superior to leg compression for managing POTS and nOH symptoms [17,19,28]. Nearly a third of total blood volume may be retained in the splanchnic mesenteric bed of the abdomen [28]. Abdominal compression helps promote the return of this volume to the heart, whereas leg compression is ineffective in targeting this volume. Abdominal compression is also preferrable as up to 69% of patients with POTS have May–Thurner syndrome, a condition where the veins draining the left leg are intrinsically compressed [29].

Existing medical leg compression and abdominal compression products, as well as shapewear, do not fully meet the needs of POTS and nOH patients, as evidenced by participant ratings on multiple domains. Overall, patients report the current commercially available products to be fairly unappealing and average with respect to ease of use, durability, cost-effectiveness, and consistency. Shapewear and abdominal compression garments are generally more concealable than leg compression garments. This is because they can fit discreetly beneath clothing and target specific areas. Leg compression garments, on the other hand, are typically designed to cover the entire leg or a significant portion thereof, making them less easily hidden. Nevertheless, leg compression garments often tend to be more comfortable than shapewear or abdominal compression garments. This is because they provide a more even distribution of pressure, reducing the likelihood of discomfort or skin irritation.

### 4.2. Compression Garments for POTS and nOH Patients

The management of symptoms in patients with POTS and nOH through compression garments has been met with limited success, with many patients reporting discomfort and poor fit with off-the-shelf compression garments. Despite almost every participant reporting trying at least one form of compression garments, less than 10% of this cohort consider compression to be an effective approach for managing symptoms. We believe a significant factor contributing to this ineffectiveness is the lack of proper product fit, which is exacerbated by the design of existing compression garments that are primarily intended for other medical conditions.

Current medical compression garments are often designed with a focus on conditions like deep vein thrombosis (DVT) or post-operative needs, which do not adequately address the unique requirements of patients suffering from POTS or nOH. For instance, leg compression garments designed for DVT typically aim to prevent blood clots. Post-surgical abdominal garments are tailored to manage edema and support healing in a specific surgical context [30]. This mismatch in design leads to garments that may not provide the necessary targeted compression or comfort for patients with POTS or nOH, who require a different pressure profile and fit to effectively manage their symptoms. This highlights the need for more targeted research and development in compression garment technology that specifically addresses the requirements of patients with autonomic dysfunctions, rather than relying on garments designed for unrelated conditions.

It is known that compressive garments must apply 30–40 mmHg of pressure to reduce venous pooling [14]. Studies have shown that many commercially available garments provide pressures that are significantly lower than the clinically recommended range, often averaging around 15–25 mmHg, which is insufficient for conditions requiring higher compression [31]. This discrepancy can lead to continued orthostatic intolerance in patients who rely on these garments for symptom management.

On the other hand, continuous abdominal compression near 40 mmHg can be bothersome to POTS patients as they have high rates of irritable bowel syndrome, gastroparesis, and GERD [7,25]. Abdominal compression is not necessary when a patient is maintaining their body position for long periods of time. Hence, an optimal product would be able to engage abdominal compression when a patient changes body position and disengage afterwards.

Moreover, the variability in individual anatomy and the specific needs of patients with POTS and nOH further complicates the effectiveness of garments. Many patients report poor fit with off-the-shelf compression garments, which can lead to non-compliance and ultimately diminish the therapeutic benefits [32]. The current market lacks sufficient options for customization, leaving many patients with ill-fitting garments that do not provide adequate support or relief. Adjustable garments could potentially improve patient outcomes significantly, as they would be tailored to the unique contours and pressure needs of the individual.

### 4.3. Compliance to Compression Therapy

Another issue affecting compliance with compression therapy is the lack of proper patient education regarding its use, which can lead to frustration and discontinuation of treatment. If healthcare providers do not specify the need for abdominal compression, patients may default to using leg compression alone, which may not adequately address their symptoms [33]. Studies have also highlighted that patients may not receive adequate guidance on how to wear these garments correctly, which can lead to inconsistent application [34]. These oversights can result in patients perceiving compression therapy as ineffective, as they may not experience the desired relief.

As a call to action, healthcare providers must prioritize patient education regarding compression therapy for POTS and nOH. This includes not only explaining the importance of abdominal compression but also providing practical guidance on how to wear and care for these garments. By fostering an environment of open communication and support, providers can help patients navigate their treatment options more effectively, leading to improved compliance and better management of their symptoms. The development of educational materials, workshops, and personalized consultations can serve as valuable resources for patients, ensuring they have the knowledge and tools necessary to succeed in their treatment journey.

### 4.4. Strengths and Limitations

To the best of our knowledge, this is the largest survey study to date aimed to understand consumer perspectives of commercial compression garments from POTS and nOH patients.

While segmentation into medical leg compression, medical abdominal compression, and shapewear can provide a general framework for understanding forms of compression products, it is important to recognize the limitations. Within each category, inter-product variability may influence patient preference based on specific features. The emergence of hybrid products and the variability in design and purpose necessitate a more nuanced perspective of these garments.

Approximately two-thirds of participants tried multiple product types. By focusing solely on the most frequently worn garment, the study may overlook insights into less frequently used garments and fail to understand garment switching. To address these limitations, further studies should ask participants to rate all types of compression garments they used and include questions about garment use and choice.

This observational study may be limited by unequal accessibility to different types of garments due to socioeconomic factors, availability, and cost. Future randomized controlled trials can provide a more robust evaluation by randomly assigning participants to intervention and control groups, blinding researchers and participants, standardizing the use of compression garments, and using clear outcome measures with long-term follow-up.

Finally, the current understanding of the role of compression therapy in managing POTS and nOH remains somewhat ambiguous, particularly regarding whether it serves as an acute or long-term relief solution. While compression garments are widely recommended for these conditions, the evidence supporting their efficacy over varying mangitudes and time frames is limited and often anecdotal. Further research is needed to investigate the efficacy of abdominal compression and leg compression during different scenarios.

Despite these limitations, this study highlights a critical gap between the use of compression garments and their corresponding efficacy for management of POTS and nOH symptoms. We propose urgent innovation in product design and patient education to address this discrepancy. By optimizing garment design, improving patient understanding of proper usage, and tailoring recommendations based on individual needs, the benefits of compression therapy for POTS and nOH patients can be enhanced.

## 5. Conclusions

Our findings indicate that there is an unmet need for a compression garment that aligns with the values of POTS and nOH patients. There is potential consumer demand for the development of adjustable compression devices that can engage and disengage quickly. Such devices would allow patients to modify the level of compression according to their comfort and activity level, thus enhancing adherence to treatment protocols. Adjustable compression systems show promise in maintaining effective pressure while accommodating the dynamic needs of patients throughout their daily activities. By allowing for quick adjustments, these devices could mitigate the discomfort associated with high tonic compression and improve overall patient satisfaction and compliance with compression therapy.

## Figures and Tables

**Table 1:** Outline of the different product features evaluated by patients for their most frequently used compression garment.

Product Feature	Description
Comfort	Are you comfortable while wearing the garment?
Aesthetic appeal	Does the garment fit into your existing wardrobe?
Concealability	Is the garment visible underneath clothing?
Ease of use	Can the garment easily be donned and doffed?
Durability	Can the garment withstand many usages?
Cost-effectiveness	Is the garment fairly priced considering its purpose?
Consistency	Does the garment reliably and consistently apply therapeutic pressure?

**Table 2:** A table comparing the demographics and existing treatment approaches of NOH and POTS patients.

	nOH (N = 28)	POTS (N = 330)	Overall (N = 358)	*p*-Value
Age (Years)				0.00011
Mean (SD)	49.6 (14.8)	36.9 (11.4)	37.9 (12.2)	
Gender				0.4317
F	26 (92.9%)	314 (95.2%)	340 (95.0%)	
M	2 (7.1%)	8 (2.4%)	10 (2.8%)	
Other	0	8 (2.4%)	8 (2.3%)	
Ethnicity				0.9231
Black or African American	0 (0%)	3 (0.9%)	3 (0.8%)	
White	27 (96.4%)	297 (90.0%)	324 (90.5%)	
Other	1 (3.6%)	30 (9.1%)	31 (8.7%)	
BMI				0.0110
Mean (SD)	24.5 (5.87)	27.7 (7.69)	27.5 (7.60)	
Tried shapewear				0.6052
N	15 (53.6%)	160 (48.5%)	175 (48.9%)	
Y	13 (46.4%)	170 (51.5%)	183 (51.1%)	
Tried abdominal compression				0.1531
N	11 (39.3%)	176 (53.3%)	187 (52.2%)	
Y	17 (60.7%)	154 (46.7%)	171 (47.8%)	
Tried leg compression				0.6287
N	1 (3.6%)	19 (5.8%)	20 (5.6%)	
Y	27 (96.4%)	311 (94.2%)	338 (94.4%)	
Most used garment				0.6909
Shapewear	7 (25.0%)	65 (19.7%)	72 (20.1%)	
Abdominal compression	5 (17.9%)	43 (13.0%)	48 (13.4%)	
Leg compression	16 (57.1%)	218 (66.1%)	234 (65.4%)	
None	0 (0%)	4 (1.2%)	4 (1.1%)	

**Table 3:** A table comparing the effectiveness of lifestyle modifications, compression, and pharmacotherapy between nOH and POTS patients.

	**nOH** **(N = 28)**	**POTS (N = 330)**	**Overall (N = 358)**	***p*-Value**
Fluid and salt intake				0.509
N	10 (35.7%)	139 (42.1%)	149 (41.6%)	
Y	18 (64.3%)	191 (57.9%)	209 (58.4%)	
Exercise				0.633
N	24 (85.7%)	258 (78.2%)	282 (78.8%)	
Y	4 (14.3%)	72 (21.8%)	76 (21.2%)	
Compression				0.7287
N	26 (92.9%)	300 (90.9%)	326 (91.1%)	
Y	2 (7.1%)	30 (9.1%)	32 (8.9%)	
Pharmacotherapy				0.9894
N	16 (57.1%)	189 (57.3%)	205 (57.3%)	
Y	12 (42.9%)	141 (42.7%)	153 (42.7%)	

## Data Availability

The data that support the findings of this study are available from the corresponding author, upon reasonable request.

## Appendix A

**Table A1:** Comparison of feature ratings between three different forms of compression garments.

	Abdominal Compression Garments	Leg Compression Garments	Shapewear	*p*-Value
Comfort				0.0299
Mean (SD)	2.94 (1.08)	3.05 (1.11)	2.67 (0.93)	
Aesthetic appeal				0.606
Mean (SD)	2.63 (1.12)	2.50 (1.09)	2.63 (0.97)	
Concealability				0.00246
Mean (SD)	2.90 (1.22)	2.91 (1.08)	3.43 (1.21)	
Ease of use				0.945
Mean (SD)	3.21 (1.03)	3.15 (1.09)	3.15 (0.93)	
Durability				0.592
Mean (SD)	3.65 (1.06)	3.52 (0.92)	3.47 (0.84)	
Cost effectiveness				0.028
Mean (SD)	3.15 (0.92)	2.73 (1.05)	2.90 (1.02)	
Consistency				0.779
Mean (SD)	3.33 (1.12)	3.35 (1.21)	3.24 (1.23)	
